# Radial Artery Occlusion Recanalization via the Distal Radial Approach Followed by Vertebral Artery Stent Placement

**DOI:** 10.7759/cureus.95408

**Published:** 2025-10-25

**Authors:** Rui Li, Min Feng

**Affiliations:** 1 Neurology, Chongqing Qianjiang Central Hospital, Chongqing, CHN; 2 Pediatrics, Chongqing Qianjiang Central Hospital, Chongqing, CHN

**Keywords:** distal radial artery, neurointerventional, radial artery occlusion, stroke, vertebral artery stent placement

## Abstract

Transradial access is increasingly utilized in neurointerventional procedures; however, due to the narrow diameter of the radial artery, puncture may induce radial artery spasm, and postoperative radial artery occlusion is a relatively common complication following transradial access. Although often asymptomatic, it poses challenges for subsequent radial artery access. In this instance, the patient experienced proximal radial artery occlusion subsequent to radial artery angiography, coupled with severe stenosis at the origin of the right vertebral artery. The patient strongly preferred radial artery access. Proximal radial artery puncture proved unsuccessful, so after puncturing the distal right radial artery, recanalization of the proximal radial artery was conducted, followed by stent placement in the right vertebral artery. The surgery was successful, and no complications arose postoperatively, offering a reference for future studies on proximal radial artery occlusion, distal radial artery access, and neurointerventional treatment.

## Introduction

Neurointerventional therapy has become one of the main treatment methods for cerebrovascular diseases[1]. Traditional neurointerventional therapy uses the femoral artery approach[2]. The radial artery approach has become the standard pathway for coronary intervention therapy[3]. The application of the radial artery approach in neurointerventional diagnosis and treatment was first reported by Cowling et al. in 1997, with only a few case reports previously [4]. With the innovation and optimization of neurointerventional therapy devices, the radial artery approach is increasingly widely used in the field of neurointervention[5]. Radial artery occlusion is a relatively common complication after transradial access (TRA), and post-puncture radial artery occlusion is the most challenging [6].

## Case presentation

**Figure 1:**
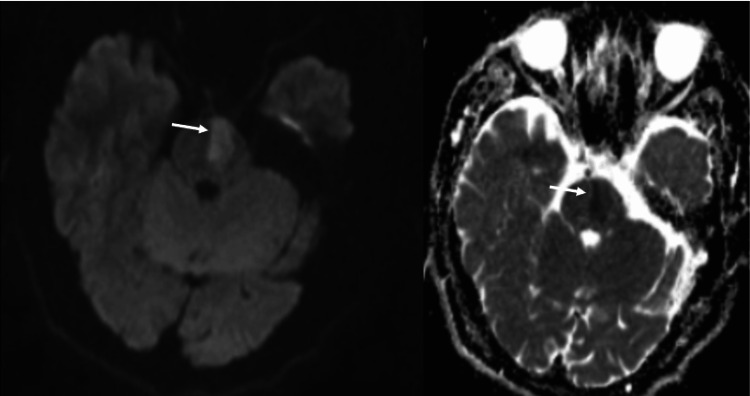
Local county hospital cranial MRI shows left pontine infarction (arrow indicated)

A 60-year-old male patient was admitted to the hospital due to right-sided limb weakness for 26 days. Twenty-six days before admission, the patient suddenly experienced right-sided limb weakness without any apparent cause, accompanied by unclear speech and left-sided deviation of the mouth corner. He sought medical attention at a local hospital, where a head CT scan did not show any bleeding, and he was treated with alteplase for intravenous thrombolysis. A head MRI suggested brainstem infarction (Figure 1).

**Figure 2:**
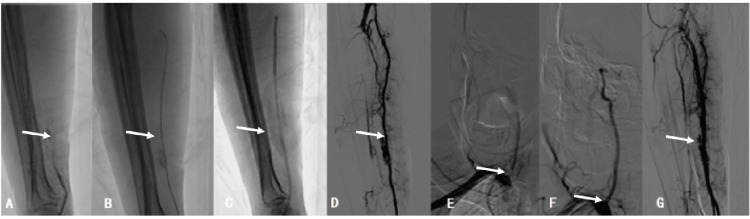
Basic surgical process. A: Right radial artery proximal occlusion suggested by vascular sheath angiography (arrow indicated). B: Introduction of a guidewire through the occluded segment to the brachial artery (arrow indicated). C: Guiding catheter being advanced to the right brachial artery and repeated aspiration (arrow indicated). D: Angiography revealing patency of the right radial artery (arrow indicated). E: Severe stenosis at the origin of the right vertebral artery (arrow indicated). F: Placement of a Bridge 4.0 mm x 16 mm stent (arrow indicated). G: Postoperative angiography revealing patency of the right radial artery (arrow indicated).

Subsequently, cerebral angiography via the right radial artery revealed severe stenosis at the origin of the right vertebral artery (Figure 2E). Following treatment with indobufen 0.1 g bid, clopidogrel 75 mg for antiplatelet aggregation, atorvastatin calcium 20 mg to regulate lipids and stabilize plaques, benzyphenate to improve collateral circulation, and rehabilitation physiotherapy, the patient showed improvement. He was admitted to our hospital on January 31, 2025, for endovascular treatment of the right vertebral artery origin. Admission physical examination revealed a temperature of 36.5℃, pulse of 85 beats/min, respiration of 20 breaths/min, and blood pressure of 130/81 mmHg. Consciousness was clear, speech was slightly unclear, and the pharyngeal reflex was normal. Memory, calculation, orientation, and comprehension were all normal. No ptosis of eyelids was observed, eyeballs moved freely, bilateral pupils were equal and round, with a diameter of 3.0 mm, and the light reflex was sensitive. Hearing was roughly normal. Heart rhythm was regular, no murmurs, bilateral lung breath sounds were clear, soft abdomen, and no tenderness. The right nasolabial fold was slightly shallow, the corners of the mouth were slightly deviated to the left, and the tongue protruded centrally. Left limb muscle strength and muscle tone were normal. Right limb muscle tone was normal, right limb muscle strength was grade IV, right limb temperature, pain, and touch sensation were slightly reduced, and pathological signs were (-). Weak pulsation of the right radial artery was observed. The National Institutes of Health Stroke Scale (NIHSS) score was 4 points (facial paralysis 1 point, right upper limb 1 point, right lower limb 1 point, sensation 1 point). The Modified Rankin Scale (mRS) score was 0 points before onset.

Additional tests, particularly out-of-hospital head MRI, indicated brainstem infarction (Figure 1). Cerebral angiography suggested severe stenosis at the origin of the right vertebral artery (Figure 2E). The diagnosis was 1) cerebral infarction (left pons, The Trial of Org 10172 in Acute Stroke Treatment classification: large artery atherosclerosis type) and 2) severe stenosis at the origin of the right vertebral artery. The patient was diagnosed with left pontine infarction, severe stenosis at the origin of the right vertebral artery, and symmetrical bilateral vertebral arteries. The treatment process was as follows: A stent placement procedure for the right vertebral artery was planned. After communicating the surgical approach with the patient and family, the patient strongly preferred the right radial artery access route. However, after puncturing the proximal right radial artery outside the hospital, the pulse was weak, making it difficult to place the catheter again in the proximal right radial artery. With the patient's and family's consent and signature, on February 7, 2025, a right distal radial artery access route was used to perform radial artery revascularization and stent placement at the origin of the right vertebral artery.

**Figure 3:**
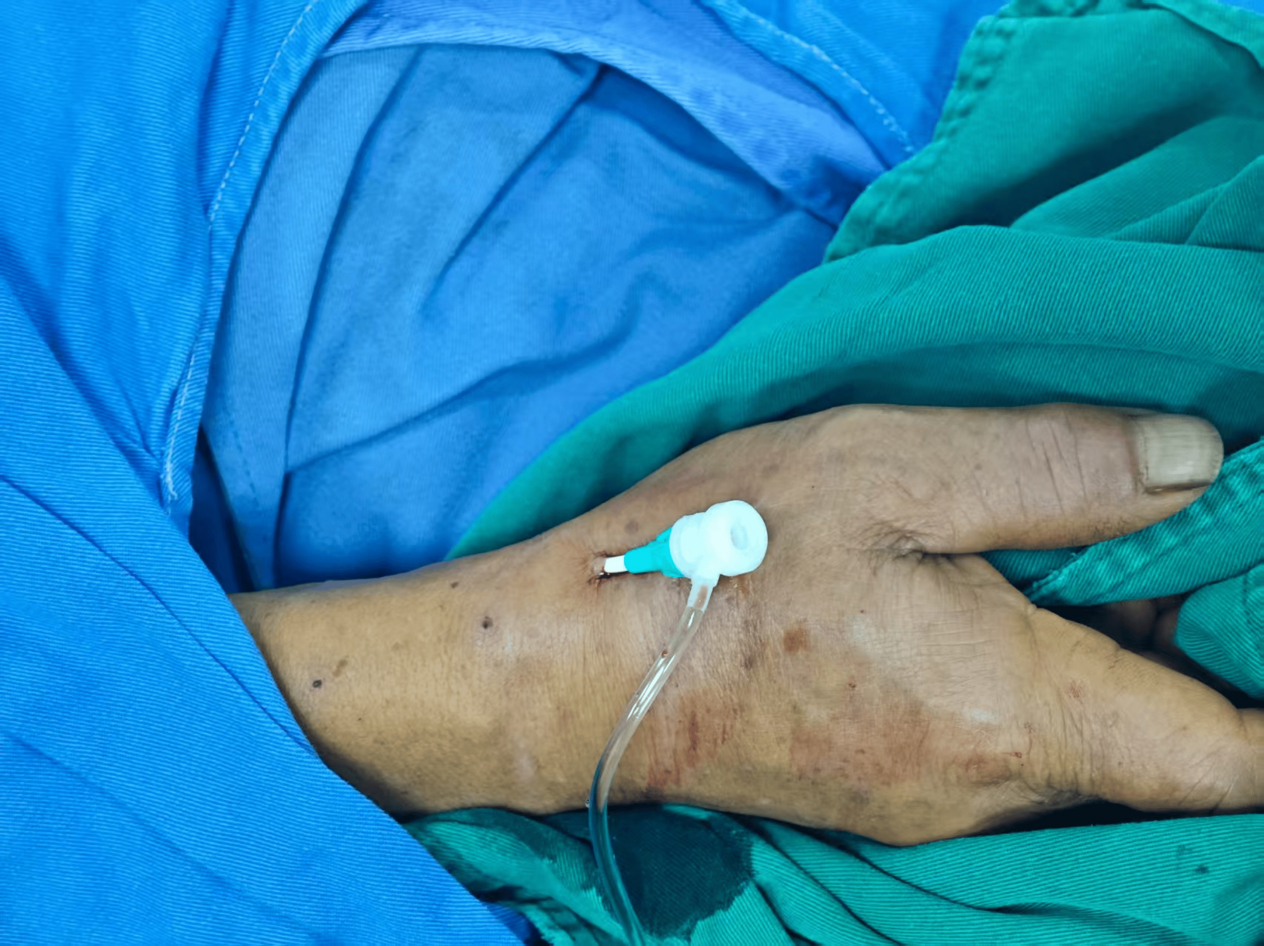
Situation of sheath placement via the distal radial artery approach (a 6F sheath is shown in the figure)

The patient was brought into the catheterization room, placed in a supine position, and disinfection was applied. Local infiltration anesthesia was performed at the puncture site using 2% lidocaine. The puncture was attempted using the Seldinger method, but the radial pulse near the proximal radial artery was weak, and repeated attempts were unsuccessful. Subsequently, puncture of the distal radial artery (snuffbox area) was performed under local anesthesia with 0.1% lidocaine (2 ml). Using a Terumo puncture needle (6F) with the Seldinger technique, the right distal radial artery was punctured, and blood return was observed after withdrawing the needle core. The sheath was slowly retracted, and good bleeding was noted. A guidewire was inserted, the sheath was removed, and a partial 6F arterial sheath was placed along the guidewire (Figure 3). Through the sheath, 200 μg of nitroglycerin and 400 U of heparin were injected. Smoke within the sheath revealed occlusion of the radial artery and a thrombus shadow locally (Figure 2A), suggesting that the occlusion time was short and the thrombus had not fully organized, so aspiration to remove the thrombus was planned. A molli guidewire passed through the occluded segment to the brachial artery (Figure 2B), and a 6F guiding catheter was advanced to the right brachial artery (Figure 2C). The molli guidewire was withdrawn, and repeated aspirations were performed, resulting in the removal of several thrombi. Angiography showed patency of the right radial artery (Figure 2D). Along the molly guidewire, a 6F guiding catheter was advanced to the right subclavian artery, and angiography revealed severe stenosis at the origin of the right vertebral artery (Figure 2E). A Bridge 4.0 mm x 16 mm stent was placed (Figure 2F), significantly improving the stenosis without residual narrowing. Post-procedure angiography of the radial artery showed patency of the radial artery (Figure 2G). After the procedure, the distal radial artery sheath was removed, and the area was compressed and bandaged with a sterile gauze to stop bleeding. The patient returned to the ward safely postoperatively, and the area was compressed and bandaged with a sterile gauze to stop bleeding.

The patient was discharged on February 9, 2025. Upon discharge examination, there was clear consciousness, slightly unclear speech, and a normal pharyngeal reflex. Bilateral pupils were equal and round, with a diameter of 3.0 mm, and a sensitive light reflex. The right nasolabial fold was slightly shallow, the corner of the mouth was slightly deviated to the left, and the tongue protrusion was central. There was normal muscle strength and tone in the left limb. There was normal muscle tone in the right limb, muscle strength grade V in the right limb, slightly reduced temperature, pain, and touch sensation in the right limb, and pathological signs (-). A palpable radial artery pulse on the right side was observed. The NIHSS score was 2 points (facial paralysis 1 point, sensory 1 point). After discharge, oral indobufen 0.1 g bid, clopidogrel 75 mg for antiplatelet aggregation, and atorvastatin calcium 20 mg for lipid regulation were continued. After multiple telephone follow-ups, the patient did not report any special discomfort, but due to family reasons, he could not attend the follow-up. Appointments in person and ongoing follow-up were recommended.

## Discussion

Neurointerventional therapy has become one of the main treatment methods for cerebrovascular diseases [1]. Vascular interventional therapy can choose different vessels for surgery, with femoral artery access being the classic approach. After femoral artery access, patients need to stay in bed for a period of time, which may increase the incidence of postoperative complications such as deep vein thrombosis in the lower extremities, pulmonary embolism, and vagus nerve reflex, while the complication rate during local puncture is also relatively high[7]. The transradial approach (TRA) is well-established within interventional cardiology and is recommended as first-choice access by the American Heart Association[8-9]. Given the associated benefits to patient safety, satisfaction, and decreased recovery time, a first-line TRA for endovascular surgery has gained traction in both peripheral and neurointerventional radiology[10-11].

With the development of interventional techniques, the safety and feasibility of radial artery access have been increasingly reported in domestic and international literature. Postoperative recovery time is shorter, patient satisfaction is higher, and the success rate of superselective procedures is higher in patients with a type III arch or horn-shaped arch[12-13]. However, radial artery access also has its own disadvantages, such as radial artery occlusion, puncture failure due to radial artery spasm, hematoma leading to compartment syndrome due to improper post-puncture compression, etc. [6]. The incidence of radial artery occlusion is 1-33%, mostly asymptomatic[14-15]. Once radial artery occlusion occurs, the same-side radial artery access surgery will face challenges. Multiple punctures of the radial artery, increased ratio of vascular sheath to radial artery diameter, lack of anticoagulation during perioperative intravascular procedures, and prolonged postoperative occlusive compression hemostasis are the main causes of radial artery occlusion[16-17]. Methods to avoid radial artery occlusion include reducing the size of sheaths and catheters, adequate intraoperative anticoagulation, non-occlusive hemostasis, with the minimum pressure force used during hemostasis compression, and compression time controlled within two hours, avoiding radial artery spasm[18].

## Conclusions

In this case, the patient underwent cerebral angiography via puncture of the right proximal radial artery outside the hospital. Postoperatively, the radial artery pulse was weak, and repeated puncture of the proximal radial artery failed. It was considered that heparin was not injected into the sheath during surgery, and the compression time was relatively long (eight hours), leading to thrombosis of the radial artery. There are few reports on recanalization of the radial artery occlusion via the distal radial artery approach followed by cerebral vascular interventional treatment. The patient underwent radial artery approach angiography outside the hospital, with good experience and high comfort. When undergoing further treatment, there was a certain rejection of the femoral artery approach and a strong preference for the radial artery approach.

Repeated recanalization of the occluded radial artery at the original site followed by neurointerventional treatment is safe and feasible. However, the puncture of the proximal radial artery failed during the operation, and the distal radial artery approach was used to recanalize the proximal radial artery and then place a stent in the right vertebral artery. The surgery was successful, and no complications occurred postoperatively. In cases of proximal radial artery occlusion, we accessed the proximal radial artery by puncturing the distal radial artery and then placed a vertebral artery stent. By analyzing the relevant diagnostic and treatment experience of this patient, we aim to provide a reference for future research on proximal radial artery occlusion and neurointerventional treatment via the distal radial artery approach.
